# Overexpression of SYF2 promotes cell proliferation and correlates with poor prognosis in human breast cancer

**DOI:** 10.18632/oncotarget.18188

**Published:** 2017-05-23

**Authors:** Feng Shi, Feng-Feng Cai, Lu Cai, Xiao-Yan Lin, Wei Zhang, Qin-Qin Wang, Yu-Jie Zhao, Qi-Chao Ni, Hua Wang, Zhi-Xian He

**Affiliations:** ^1^ Department of General Surgery, Affiliated Hospital of Nantong University, Nantong University, Nantong, PR China; ^2^ Department of Breast Surgery, Yangpu Hospital, Tongji University School of Medicine, Shanghai, PR China

**Keywords:** SYF2, breast cancer, proliferation, prognosis

## Abstract

SYF2, a known cell cycle regulator, is reported to be involved in cell cycle arrest by interacting with cyclin-D-type binding protein 1. In the present study, we investigated the role of SYF2 in human breast cancer (BC) progression. SYF2 was highly upregulated in BC tissues and cell lines, as per Western blot and immunohistochemistry analysis. The SYF2 expression level had a significant correlation with the tumor grade and Ki-67 expression. *In vitro* starvation-refeeding experiment and SYF2-siRNA transfection assay demonstrated that SYF2 could promote proliferation of BC cells, while SYF2 knockdown resulted in cells cycle arrest at G1/S phase, reducing the cell growth rate of BC cells. These results indicated that SYF2 promotes human BC progression by accelerating the BC cells’ proliferation. SYF2 could be a novel therapeutic target in human BC therapies.

## INTRODUCTION

Breast cancer remains the most common cancer and is rated second in cancer related mortality (World Cancer Report, 2014). Although substantial advances in surgical techniques and chemotherapeutic treatments have been made in BC, the prognosis remains unfavorable, mainly attributed to late diagnosis and limited management options [1]. Currently, a promising strategy for breast cancer is the development of novel therapeutic targets, many of which have been studied extensively in preclinical and clinical models [2]. Trastuzumab, for example, is the first anti-HER2 (human epidermal growth factor receptor 2) monoclonal antibody applied in systemic adjuvant therapy for patients with HER2-positive breast cancer, with significant benefits in BC treatment [3]. Therefore, the identification of the molecular markers for prognosis is important in benefiting BC patients.

SYF2, also known as p29 CCNDBP1 interactor [4], is a chromosome-associated protein [5], initially identified associated with Grap2 cyclin D interacting protein (GCIP) in yeast two-hybrid screening and *in vitro* GST-binding assay [4, 6]. One report has shown that GCIP correlation with cyclin D1 (considered as a SYF2 interacting protein) is dysregulated in several tumor entities, e.g. colon cancer, prostatic cancer, ovarian cancer [7]. Previous studies showed that SYF2 (synthetic lethal with CDC forty protein 2) is mainly involved in cell cycle, modulating transcriptional and posttranscriptional control mechanisms of α-tubulin [8, 9], pre-mRNA splicing [10], and DNA repair [5, 11]. However, the role of SYF2 in BC genesis has not yet been elucidated.

In order to verify the role of SYF2 in breast cancer, we performed a series of experiments and found that SYF2 expression was upregulated in BC specimens and BC cell lines. We also confirmed the positive correlation of SYF2 expression with Cyclin D1 and cell proliferation of breast cancer cells. In addition, we transfected BC cell line with siRNA. As expected, we found that knockdown of SYF2 gene could inhibit BC cell proliferation. These results implied that SYF2 may be a novel prognostic marker and play a potential role in anti-proliferative therapy of breast cancer.

## RESULTS

### The expression of SYF2 in BC tissues and BC cell lines

**Figure 1:**
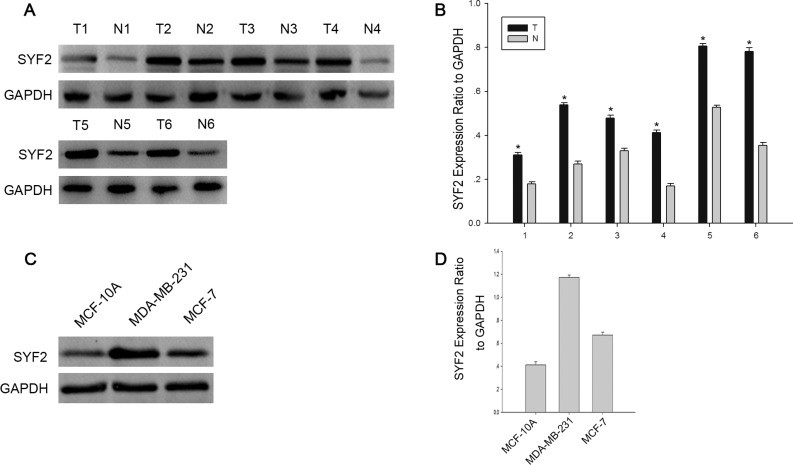
The expression of SYF2 in bresat cancer tissues and cells (**A**) Western blot analysis showing the SYF2 expression in six representative paired BC tissues(T) and adjacent normal tissues(N). (**B**) The densitometry of SYF2 normalized *to GAPDH* displayed by bar chart. The data are mean ± SEM of three independent experiments. (*P < 0.05, tumor tissues compared with adjacent nontumorous). (**C**) The expression of SYF2 in three human BC cell lines was detected by Western blot. (**D**) The bar graph displayed the ratio of the SYF2 protein to GAPDH by densitometry in the two breast cancer cell lines.

To investigate the role of SYF2 in BC, we performed Western blot analysis with six paired surgical specimens and two BC cell lines including MDA-MB-231 and MCF-7. As expected, higher expression of SYF2 was found in BC tissues compared with adjacent normal breast tissues (Figure 1A and 1B). Moreover, SYF2 was highly expressed in BC cell lines, especially in MDA-MB-231 cells. (Figure 1C and 1D).

**Figure 2:**
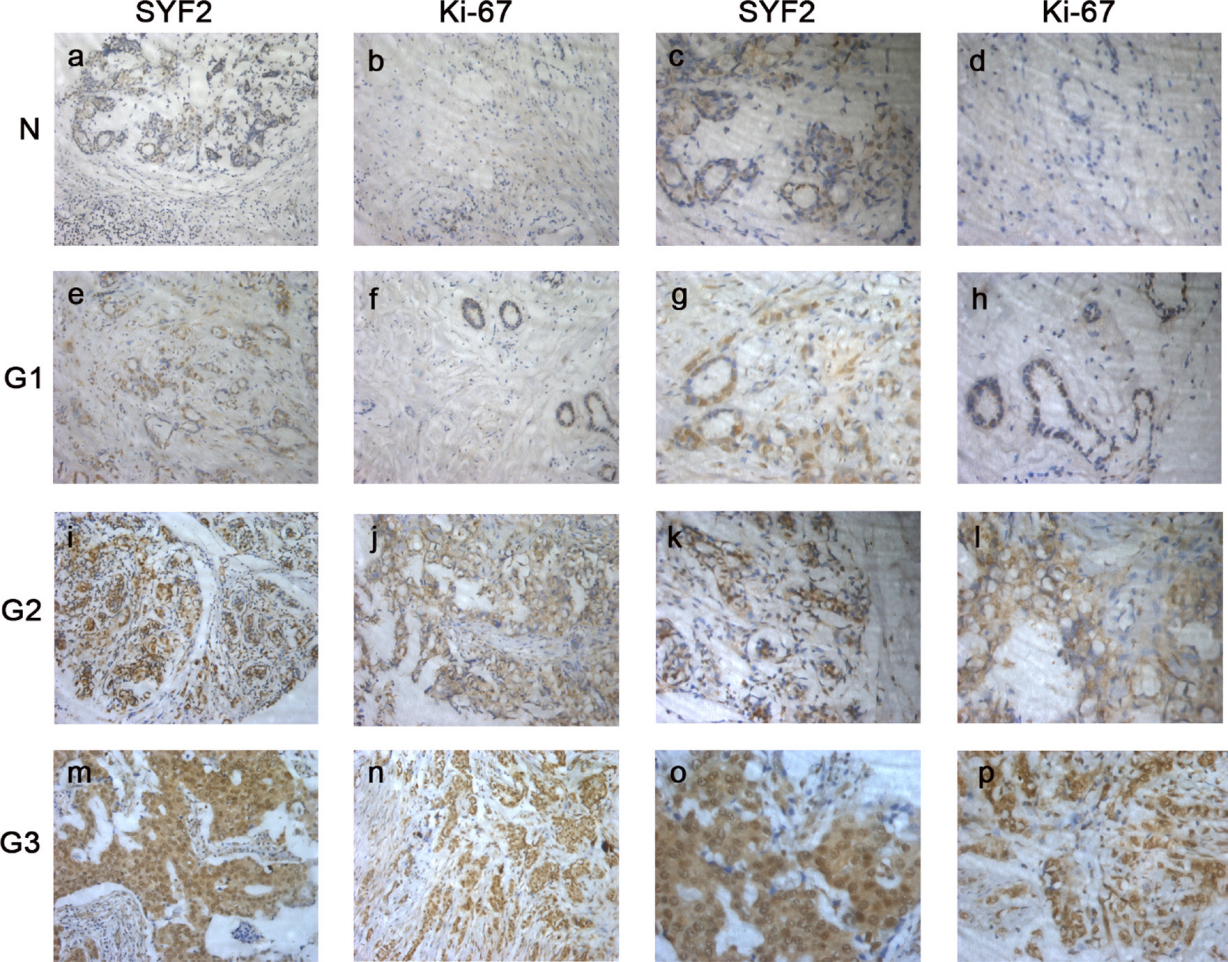
The paraffin-embedded BC tissues were stained with antibodies for SYF2 and Ki-67 and counterstained with hematoxylin (detailed in the “Materials and methods” section) (**A**–**D**) Immunoreactivity of SYF2 and Ki-67 in adjacent nontumorous breast tissue. (**E**–**H**). Immunoreactivity of SYF2 and Ki-67 in BC tissue of G1. (**I**–**L**) SYF2 and Ki-67 staining in cancer tissue of G2. (**M**–**P**) SYF2 and Ki-67 staining in cancer tissue of G3.a, b, e, f, i, j, m, n. Images in ×200 magnification.c, d, g, h, k, l, o, p. Images in ×400 magnification.

Further on, we investigated the expression of SYF2 and Ki-67 in 123 BC specimens by immunohistochemistry. As shown in Figure 2, we found that SYF2 and Ki-67 were mainly located in the nucleus of BC cells. Furthermore, the expression of SYF2 was positively correlated with Ki-67 and tumor grade.

### Correlation of SYF2 expression with clinicopathologic parameters in BC

**Figure 3:**
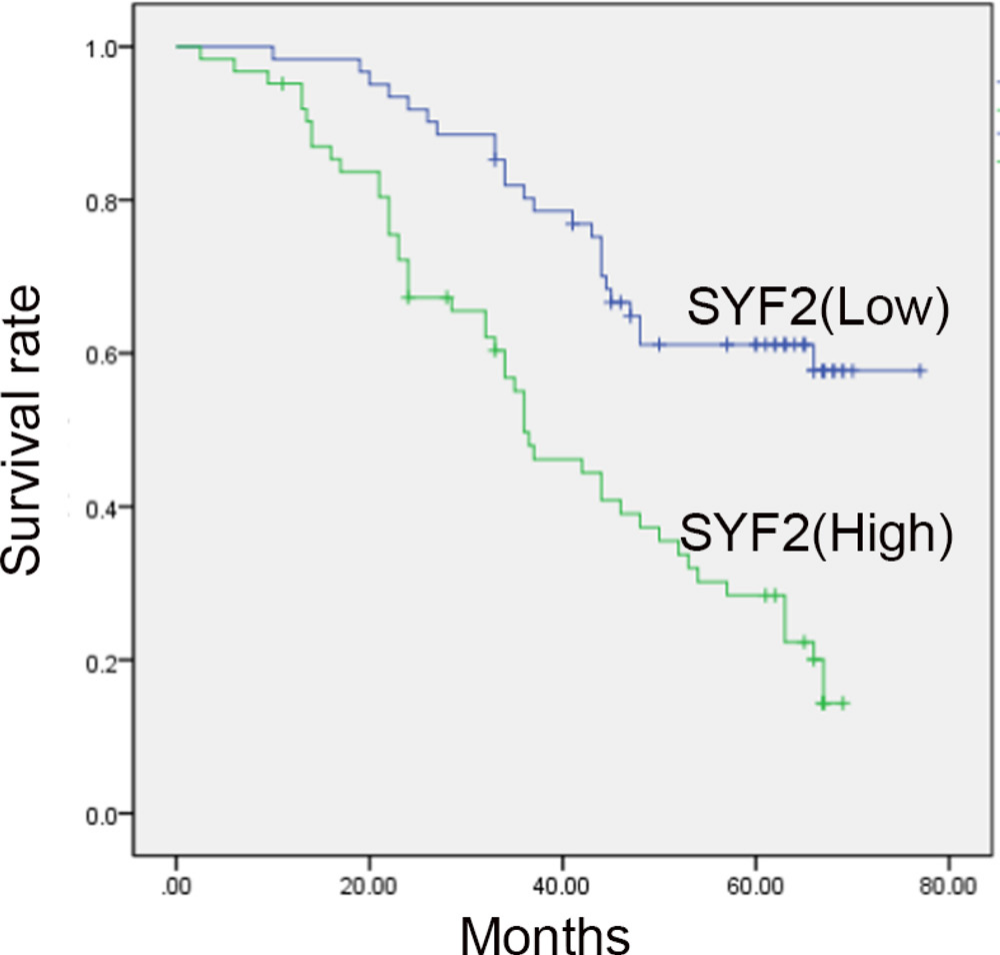
Kaplan-Meier survival analysis for high SYF2 expression versus low SYF2 expression in 123 patients of BC Patients with high expression of SYF2 had worse prognosis.

To evaluate the clinicopathological significance of SYF2, the correlation between SYF2 expression and clinicopathological parameters were estimated by Pearson χ2 test (Table 1). For statistical analysis, we divided the tumor specimens into high expression group and low expression group by the cutoff value mentioned in the “Materials and methods” section. As shown in Table 1, SYF2 expression was significantly correlated with the histologic grade (*P* = 0.012), lymph node status (*P* = 0.017) and Ki-67 (*P* = 0.004). There was no statistical correlation between SYF2 expression and other prognostic factors. Survival analysis curve was constructed to investigate the correlation between survival status and clinicopathological parameters. The results clarified that the histological grade (*P* = 0.024), lymph node status (*P* = 0.009), Ki-67 expression (*P* = 0.023) and SYF2 expression (*P* < 0.001) were substantially associated with the patients’ overall survival (Table 2). Furthermore, Cox’s proportional survival analysis revealed that the histological grade (*P* = 0.022), lymph node status (*P* = 0.003), SYF2 expression (*P* < 0.001) and Ki-67 expression (*P* < 0.001) were independent prognostic indicators for the overall survival (Table 3). Kaplan-Meier analysis was performed to determine the impact of SYF2 expression level on patients’ survival time. The survival curves showed that patients with high expression had a significantly poorer overall survival (Figure 3).

**Table 1:** Correlation between SYF2 expression and the clinicopathologic features of breast cancer

Criteria	No. case	SYF2 Expression		*P* value^a^	*χ*^2^
		Low	High		
Age					
< 50	59	33	26	0.177	1.822
≥ 50	64	28	36		
Tumor size					
≤ 2.5	23	10	13	0.515	0.423
> 2.5	100	51	49		
Histology					
Ductal	102	44	58	0.002*	9.962
Others	21	17	4		
Grade					
I	17	6	11	0.012*	8.793
II	79	47	32		
III	27	8	19		
Axillary lymph node status					
N0	45	33	12	0.017*	5.697
Nx	78	28	50		
ER					
Negative	55	25	30	0.409	0.682
Positive	68	36	32		
PR					
Negative	53	25	28	0.640	0.219
Positive	70	36	34		
HER-2					
Negative (0∼1+)	49	24	25	0.912	0.012
Overexpressed (2∼3+)	74	37	37		
Ki-67					
Low	21	17	5	0.004*	8.211
High	102	44	57		

^a^Statistical analyses were performed by the Pearson *χ*^2^ test.

*P < 0.05 is considered significant.

**Table 2:** Correlation between SYF2 expression and the clinicopathologic features of breast cancer

Criteria	No. case	Survival Status		*P* value^a^	χ^2^
		Alive	Dead		
Age					
< 50	59	29	30	0.660	0.194
≥ 50	64	34	30		
Tumor size					
≤ 2.5	23	11	12	0.718	0.130
> 2.5	100	52	48		
Histology					
Ductal	102	51	51	0.356	0.551
Others	21	12	9		
Grade					
I	17	7	10	0.024*	7.469
II	79	54	25		
III	27	12	15		
Axillary lymph node status					
N0	45	30	15	0.009*	6.777
Nx	78	33	45		
ER					
Negative	55	25	30	0.734	0.115
Positive	68	38	30		
PR					
Negative	53	21	32	0.025*	5.013
Positive	70	42	28		
HER-2					
Negative (0∼1+)	49	30	19	0.071	3.263
Overexpressed (2∼3+)	74	33	41		
Ki-67					
Low	21	6	15	0.023*	5.199
High	102	44	57		
SYF2					
Low	61	41	20	0.000*	12.390
High	62	22	40		

^a^Statistical analyses were performed by the Pearson *χ*^2^ test.

*P < 0.05 is considered significant.

**Table 3:** Contribution of various potential prognostic factors to survival by Cox regression analysis in 123 breast carcinomas specimens

	Hazard Ratio	95% confidence interval	*P*
Age	1.052	0.715–1.549	0.798
Grade	1.175	1.085–1.472	0.022*
ER	0.773	0.470–1.270	0.309
PR	0.922	0.578–1.473	0.735
CrebB2	1.170	0.765–1.791	0.469
Tumor size	0.569	0.328–0.986	0.044*
Axillary lymph node status	1.897	1.238–2.909	0.003*
Histology	2.078	1.198–3.604	0.009*
Ki-67	4.246	2.342–7.699	0.000*
SYF-2	2.610	1.711–3.981	0.000*

^a^Statistical analyses were performed by the Pearson *χ*^2^ test.

*P < 0.05 is considered significant

### SYF2 in the cell cycle progress of BC cells

**Figure 4:**
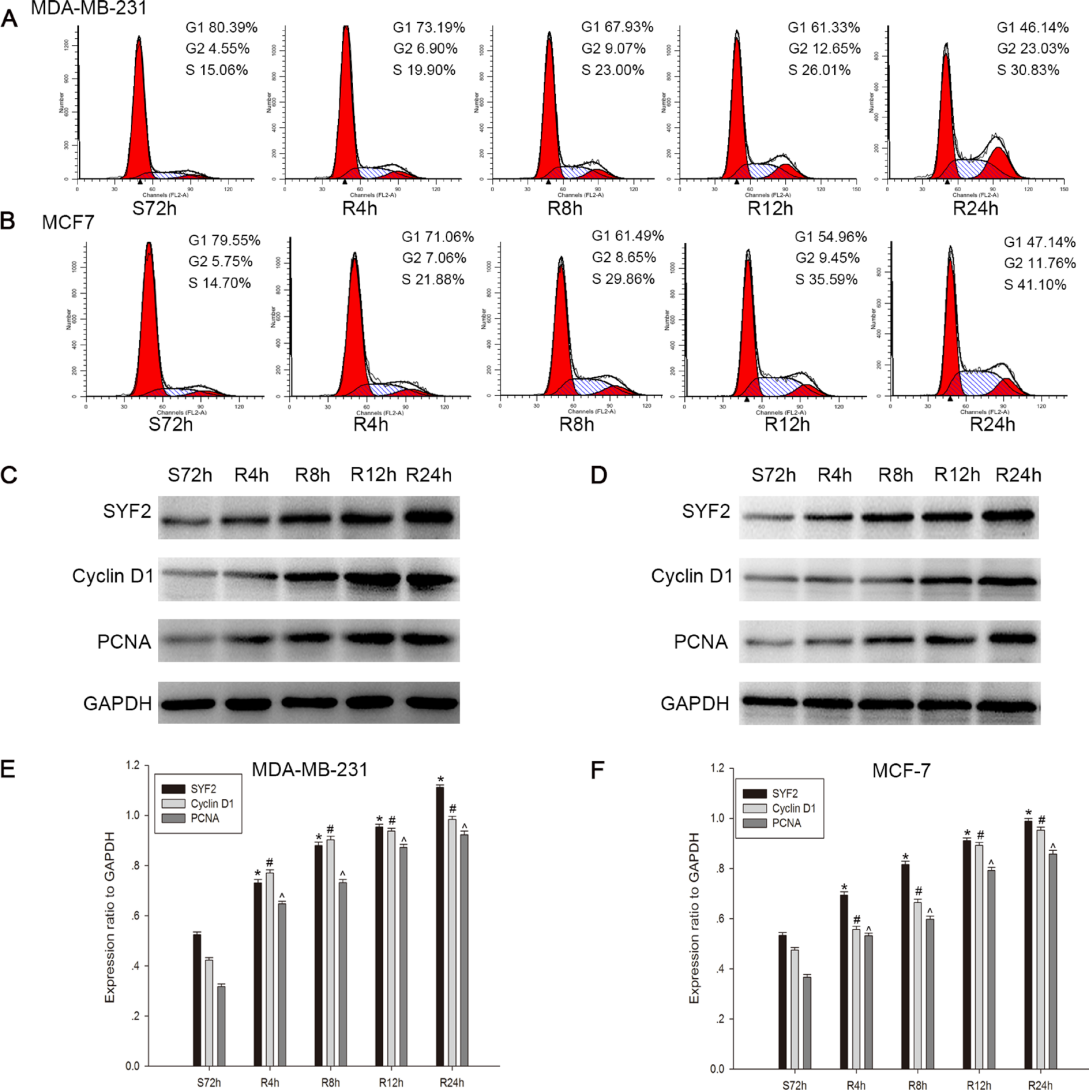
Correlation between SYF2 expression and BC cell cycle progress (**A**, **B**) Flow cytometric quantitation of cell proliferation in MDA-MB-231 cells. The cells after serum starvation for 72 h and then addition of culture medium containing 10% FBS at the indicated time points (0, 4, 8, 12, 24 h). (**C**, **D**) The cell lysates were prepared and examined by Western blotting with a series of cell-cycle-related antibodies including PCNA and cyclin D1. (**E**, **F**) The histograms below demonstrated the ratio of SYF2, PCNA, and cyclin D1 protein to GAPDH(loading control) for each indicated time point by densitometry. Date were mean ± SEM of three independent experiments. (*n =* 3, *^#^^*P* < 0.05, compared with control cells serum-starved for 72 h).

Since SYF2 was reported to be relevant for cell cycle progression by modulating transcriptional and posttranscriptional control of α-tubulin and cycline D1 [6, 8, 14], we postulated that SYF2 expression would have a similar proliferative effect on BC cells. To verify this hypothesis, we made a cell serum starvation and releasing model in MDA-MB-231 and MCF7 cells. Flow cytometry analysis showed that the two cell lines both cultured with serum free medium for 72 h were arrested at the G0/G1 phase. After serum re-addition, the MDA-MB-231 cells were released from the G1 phase (Range of 80.39 to 46.14%, Figure 4A) and reentered the S phase (Range of 15.06 to 30.83%, Figure 4A). The same results were obtained for the MCF7 cells (release from the G1 phase (Range of 79.55 to 47.14%, Figure 4B), reentering the S phase (Range of 14.70 to 41.10%, Figure 4B)). Subsequently, Western blot was applied to analyze the expression of SYF2 during cell cycle G1/S-phase transition, as well as the markers of proliferation cycline D1 and PCNA [15]. As expected, we found that SYF2 was substantially increased 4 h after serum re-addition, as were PCNA and Cycline D1 (Figure 4C and 4E; 4D and 4F). These results indicated that SYF2 may have a positive influence on the proliferation of BC in cell-cycle-dependent pathway.

### Knockdown of the SYF2 expression inhibits the BC Cells proliferation

**Figure 5:**
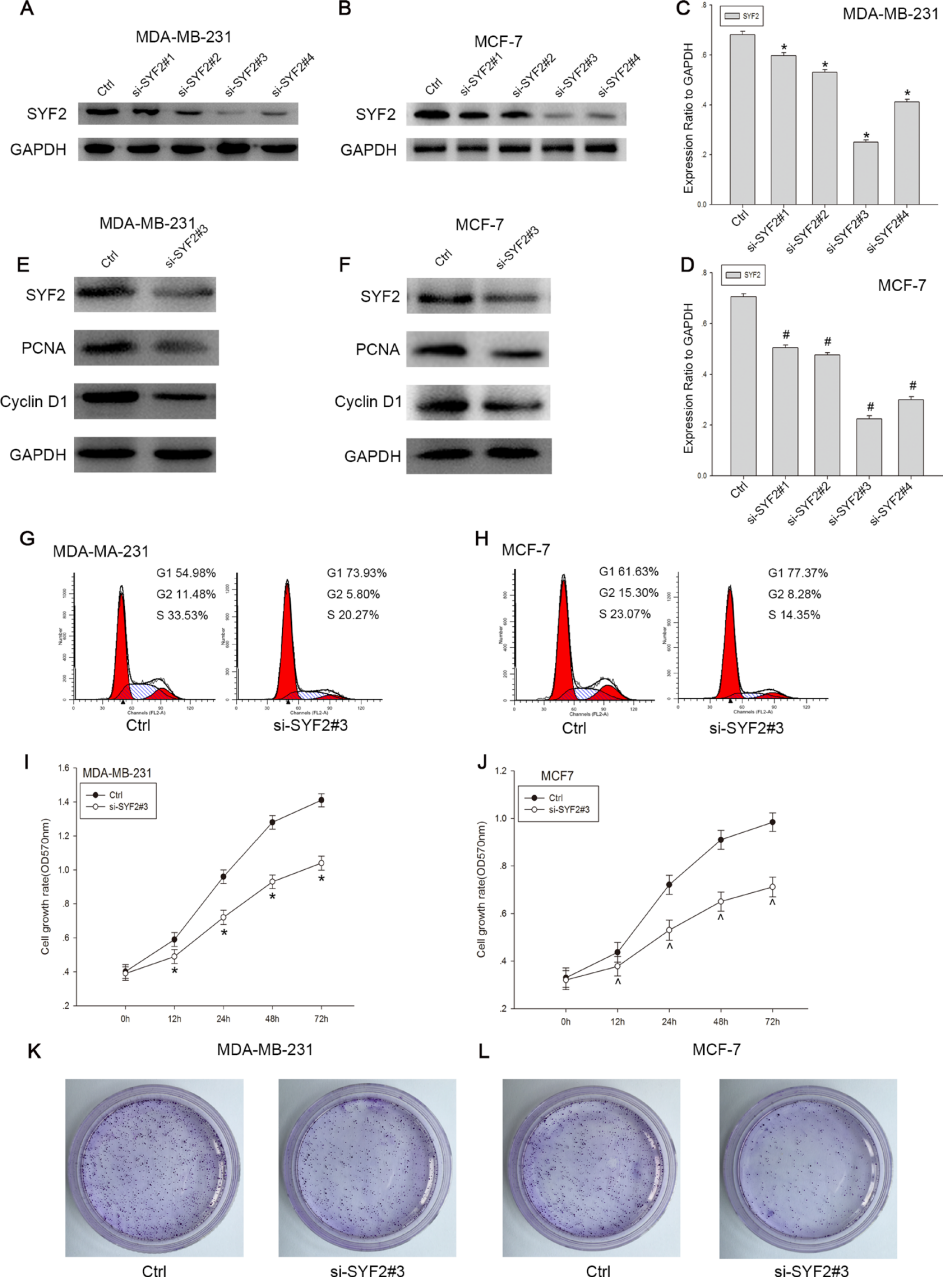
Knockdown of SYF2 inhibited proliferation of the breast cancer cell lines (**A**, **B**) To further define the effect of SYF2 on cell proliferation, we transfected the MDA-MB-231 and MCF7 cells with SYF2 siRNAs to knockdown endogenous SYF2. We also constucted a control group using negative control siRNA. SYF2 expression levels were detected by Western blot 48 h after siRNA transfection. The results showed that the si-SYF2#3 had the most dramatic decline of SYF2 expression than other three siRNAs tested. Therefore, SYF2-siRNA#3 was used for the further experiments. (**C**, **D**). The histogram below demonstrated the ratio of SYF2 protein to GAPDH by densitometry. Data were presented as mean ± SD (^*#^*P* < 0.05 compared with the control group). (**E**, **F**) Western blot analysis of cyclin D1 and PCNA in MDA-MB-231 and MCF7 cells transfected with SYF2 si-SYF2#3 and negative control siRNA. Data were expressed as means ± SD. ^*^^*P* < 0.05, compared with the control. (**I**, **J**) CCK-8 assay was performed to measure cell proliferation treated with SYF2-siRNA#3 and control siRNA. The data are mean ± SEM (*P* < 0.05, compared with control cells). (**G**, **H**) Flow cytometric analysis of cell cycle distribution 48 h later following control siRNA and si-SYF2#3 transfection. The data are mean ± SD (^*#^*P* < 0.05 compared with the control). (**K**, **L**) Colony formation analysis of control and si-SYF #3-transfected breast cancer cells.

To further confirm the vital role of SYF2 on cell proliferation in BC, MDA-MB-231 and MCF7 cells were transfected with SYF2 siRNAs and negative control. 48 h after transfection, we found that SYF2 siRNAs (SYF2-si-SYF2#1, SYF2-si-SYF2#2, SYF2-si-SYF2#3) could knock down the endogenous SYF2 compared to the negative control, and SYF2-si-SYF2#3 significantly inhibited the expression of SYF2 in breast cancer cells (Figure 5A and 5C; Figure 5B and 5D). Therefore, SYF2-si-RNA#3 was used for further experiments. SYF2-si-RNA#3 was transfected to silence the expression of SYF2 in MDA-MB-231 and MCF7 cells. After SYF2 down-regulation, the expression of PCNA and cyclin D1 were concomitantly decreased (Figure 5E, 5F). Furthermore, CCK-8 assay, Colony formation analysis and flow cytometry also suggested that downregulation of SYF2 level caused G1/S phase arrested and cell proliferation inhibition, compared with the negative control. (Figure 5G–5L). Thus, we concluded that SYF2 may play an important role in regulating breast cell proliferation in a cell cycle-dependent pathway.

## DISCUSSION

Breast cancer is the most common malignancy and the second leading cause of death from cancer in women [16]. Despite advances in various methods, including genetic, pathologic, and imaging analysis, effective detection and *appropriate* therapy in breast cancer remains a considerable challenge. In terms of genomic analysis, new techniques’ application and genomic data mining help to develop new targets for therapy and represent the primary future directions of breast cancer treatment [17]. Further research is needed to understand the molecular basis of BC as to advance in precise diagnosis and effective treatment modalities. In the present study, we demonstrate the potential role of SYF2 expression in breast cancer progression.

**Figure 6:**
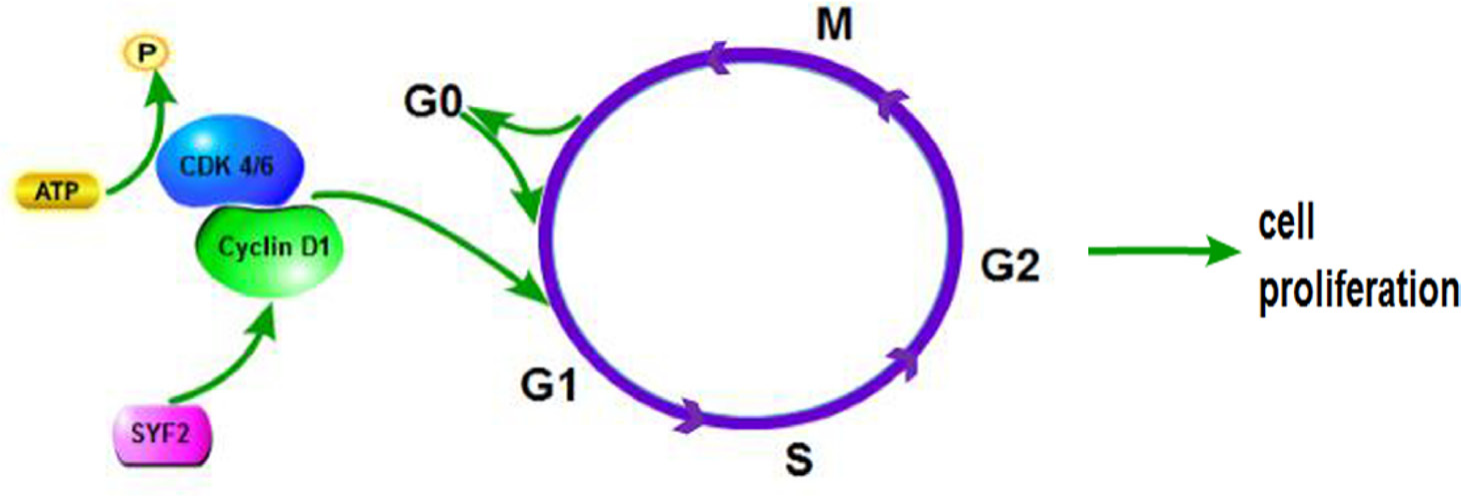
The proposed molecular mechanisms of SYF2-mediated cell proliferation in human breast cancer cells

As a yeast homolog of p29, SYF2 was initially considered as a GCIP interacting protein, which is reported to interact with cyclin D1. Overexpression of GCIP downregulates the transcriptional activity of cycline D1 promoter and thus the expression of cycline D1 [7, 18]. It is known that cycline D1, together with cycline-dependent kinase (CDK)-4 and -6, induces the transition of G1-to-S phase to promote cell proliferation [19, 20] (Figure 6). It is conceivable to hypothesize that that there is a specific interaction between GCIP, cycline D, and SYF2. SYF2 expression was also shown correlated with cell proliferation and apoptosis by mediating the expression of cycline D1 after LPS injection [14].

Recent evidence implicates that SYF2 participates in progress of diverse tumor entities, such as liver cancer [21], ovarian cancer [22], lung cancer [23], and esophageal cancer [24]. However, the role of SYF2 in breast cancer development remains obscure. We postulated that SYF2 might have a significant impact on the development of breast cancer in a cell cycle-dependent pathway [25].

Firstly, our study revealed that SYF2 was overexpressed in breast cancer tissues and breast cancer cells in both Western blot and immunohistochemistry analysis. SYF2 expression level was correlated with the histological grade and the prognosis of breast cancer. Besides, there was a positive correlation between SYF2 and Ki-67 expression, which was defined as one of prime biomarkers to reflect cell proliferation. Then, Kaplan–Meier survival analysis showed that high expression of SYF2 had a significant association with poor prognosis after surgical resection in all breast cancer patients. With these results, we demonstrated that SYF2 promoted the cell proliferation of breast cancer by serum starvation-release experiment. Using CCK-8 assay and Colony formation assays, we observed that SYF2 knockdown by siRNA could downregulate the expression of PCNA and cycline D1 in MDA-MB-231 cells. Flow cytometry analysis demonstrated that SYF2 participated in cell cycle of BC cells, and SYF2 knockdown resulted in the arrest of cell cycle at G0/G1 phase.

In summary, we confirmed that SYF2 was upregulated in breast cancer tissues and cells, and there was a positive correlation between SYF2 expression and proliferation of breast cancer. Conversely, knocking down of SYF2 results in cell cycle arrest and cell growth inhibition of BC. Based on these experimental data, it is now possible to strongly assume that SYF2 might potentially be a novel tumor marker or even molecular target for the treatment of breast cancer.

This study has several limitations that must be acknowledged and further investigated. Firstly, it is a retrospective study of 123 patients with a high attrition rate. Future prospective studies in a larger patient population are necessary to evaluate the prognostic value of SYF2 in breast cancer. As we have mentioned previously, SYF2 might play a pro-apoptotic role in neuronal apoptosis [14]. Additionally, it has been described that SYF2 can down-regulate the sensitivity of ESCC cells for cisplatin [24] and induce doxorubicin resistance in HCC cells [21]. Thus, SYF2 might have a similar impact on breast cancer cells. Further research is warranted.

## MATERIALS AND METHODS

### Patients and tissue samples

All BC specimens were obtained from patients who underwent surgical tumor resection between 2006 and 2012 at the Department of General Surgery, Affiliated Hospital of Nantong University. All patients were followed up for 1to 60 months. The study was authorized by the Ethics Committee of Affiliated Cancer Hospital of Nantong University. The samples were fixed in 10% formalin and embedded in paraffin for histopathologic diagnosis and immunohistochemical analysis. Fresh samples were immediately frozen in liquid nitrogen after surgical removal and maintained at –80°C until use. All resected specimens were classified according to the TNM classification system [12]. The clinical features of the patients, including age, histologic grade, tumor size, metastasis, histology, as well as ER, PR and Her2 status, are shown in Table 1. The median age of the patients was 47 years (range: 27–79 years). Histologic grades were defined as well-differentiated (grade I, *n =* 17), moderately differentiated (grade II, *n =* 79), and poorly differentiated (grade III, *n =* 27). The majority of tumors (*n =* 102, 84.29%) were infiltrating ductal carcinomas; the remaining 21 cases were of other types. Details are shown in Table 1.

### Antibodies

Antibodies used in this study were as follows: (1) mouse anti-SYF2 monoclonal antibody (Santa Cruz Biotechnology, USA), (2) mouse anti-human Ki-67 monoclonal antibody (Santa Cruz Biotechnology, USA), (3) mouse anti-human PCNA monoclonal antibody (Santa Cruz Biotechnology, USA), (4) rabbit antihuman cycline D1 polyclonal antibody (Santa Cruz Biotechnology, USA), (5) anti-E-cadherin (Santa Cruz Biotechnology, USA), and (6) rabbit anti-human GAPDH polyclonal antibody (Santa Cruz Biotechnology, USA).

### Western blot

Western blot analysis was done as previously described [13]. Flesh frozen tissue and cell protein were promptly homogenized in a homogenization buffer containing 1 M Tris HCl pH 7.5, 1% Triton X-100, 1% Nonidet p-40 (NP-40), 10% sodium dodecyl sulfate (SDS), 0.5% sodium deoxycholate, 0.5 M EDTA, leupeptin 10 μg/mL, aprotinin 10 μg/mL, and 1 mM PMSF, and then centrifuged at 10,000 × g for 30 min to collect the supernatant liquid. Protein concentrations were determined with a Bio-Rad protein assay (Bio-Rad, Hercules, CA, USA). Before gel electrophoresis, the supernatant diluted in 2×SDS loading buffer and boiled for 15 min. An equivalent amount of protein from each sample was electrophoresed by 10% sodium dodecyl sulfate-polyacrylamide gel electrophoresis (SDS-PAGE) and transferred to Polyvinylidene fluoride (PVDF) membrane (Immbilon; Millipore). Membranes were first blocked in 5% dried skim milk in TBST (150 mM NaCl, 20 mM Tris, 0.05% Tween 20) for 2 h, then incubated with the primary antibodies for 2 h at room temperature, washed with TBST three times, 5 min each, and then incubated with horseradish-peroxidase-linked IgG as secondary antibodies for 2 h at room temperature. After 3 washes of 15 min, the membranes were visualized using the ECL (enhanced chemiluminescence) detection systems (Pierce, Rockford, IL, USA). The densities of bands were compared using ImageJ (NIH). The experiments were carried out on three separate occasions.

### Immunohistochemical staining

Serial sections of four micrometer were prepared on glass slides, dewaxed in xylene and rehydrated in graded ethanols, then heated to 121°C in an autoclave for 3 min to retrieve the antigen using citrate buffer (pH 6.0). After rinsing in PBS (pH 7.2), the sections were incubated with a monoclonal mouse anti-SYF2 antibody (diluted 1:150) and a monoclonal mouse anti-Ki-67 (diluted 1:400) antibody overnight at 4°C. All slides were processed using the peroxidase-antiperoxidase method (Dako, Hamburg, Germany), according to the manufacturer’s instructions. All slides were counterstained with hematoxylin, dehydrated, and coverslipped. All immunostained sections were evaluated by observers blinded to the clinical and pathological characteristics of the patients.

### Immunohistochemical evaluation

All stained sections were evaluated in a blinded manner. Five high-power fields in each slide were selected randomly; at least 500 cells were counted per field to evaluate the SYF2 immunoreactivity. In half of the samples, staining was repeated two times to avoid technical errors. For assessment of SYF2 expression, intensity of immunostaining was assessed and scored as follows: 0, negative staining; 1, weak staining; 2, moderate staining; and 3, strong staining. The percentage of positive tumor cells was scored as follows: 0, < 5% tumor cells stained; 1, 6–25%; 2, 26– 50%; 3, 51–75%; and 4, > 75%. Then, the two scores were combined and a score of 0 was considered negative, 2–3 was considered weak, 4–5 was considered moderate, and 6–7 was considered strong. For statistical analysis, 0–3 were counted as low expression, and 4–7 were counted as overexpression. As for statistical analysis of Ki- 67, a cutoff value was used to distinguish tumors with low (< 50%) or high (≥ 50%) level of Ki-67 expression.

### Cell culture and cell cycle analysis

MCF-10A, MDA-MB-231 and MCF-7 were all the human BC cell lines used (Department of Oncology, Affiliated Cancer Hospital of Fudan University). All cell lines were cultured in Dulbecco’s modified Eagle’s medium (DMEM) (Gibco BRL, Grand Island, NY) supplemented with 10% heatinactivated foetal bovine serum (FBS), 2 mM L-glutamine, and 100 U/ml of a penicillin–streptomycin mixture (Gibco BRL) at 37°C and 5% CO_2_.

For cell cycle analysis, cells were fixed in 70% ethanol overnight at –20°C, incubated with 1 mg/mL RNase A for 30 min at 37°C, stained with propidium iodide (PI, 50 mg/mL, Becton–Dickinson, San Jose, CA) in PBS, and 0.5% Tween 20. The analysis was performed using a Becton Dickinson flow cytometer BD FACScan (San Jose, CA). Gating was set to exclude cell debris, cell doublets, and cell clumps. The results were representative in three independent experiments.

### siRNAs and transfection

Four siRNAs targeting SYF2 gene (Gene ID: NM_207170) sequences were as follows: 5′-TTATAATGATGATGCAGAT-3′, 5′-TAAATTAAATCACCAGGAA-3′, and 5′-GGAATGAAGCTCGTAAATT-3′, 5′-CGGAATGAAGCTCGTAAAT-3′. The negative control (Ctrl) siRNA sequence was 5′-UUCUCCGAACGUGUCACGUTT -3′, MDA-MB-231 and MCF7 cells were plated the day before transfection at 50–70% confluency. The transfections of SYF2 siRNAs were erformed with Lipofectamine 2000 (Invitrogen). The cells were collected after 48 h for further assays.

### Colony formation assays

MDA-MB-231 and MCF7 cells were seeded at a density of 200 cells/well in six-well culture plates after si-RNA transfection based on the manufacturer’s instructions and cells were cultured for 10 days. The surviving colonies (≥ 50 cells/colony) were analyzed after 0.5% crystal violet stain.

### Cell proliferation assay

Cell proliferation was measured by Cell Counting Kit-8 (Dojindo, Kumamoto, Japan) assay in accordance with the manufacturer’s instructions. In brief, the cells were seeded into a 96-well cell culture cluster at a density of 2 × 10^4^ cells/well in a volume of 100 μL and incubated overnight. 10 μL of CCK-8 reagents were added into each well, and the cells were cultured for 2 h at 37°C. The results were evaluated by an automated plate reader at 490 nm. The experiment was repeated three times.

### Ethics statement

The ethics review board approved the study design a priori. The protocol was approved by the Ethics Committee of the Department of General Surgery, Affiliated Hospital of Nantong University. Written informed consent was obtained from each patient. The method described in this study, including acquisition of blood samples, was carried out in accordance with the approved guidelines and regulations.

### Statistical analysis

The SPSS 17.0 statistical software was used for statistical analysis. The χ2 test was performed to analyze the correlations between SYF2 and Ki-67 expression and clinicopathological parameters. The Kaplan-Meier curves were calculated for the survival data analysis, and the log-rank test was performed. Multivariate analysis was performed with Cox’s proportional hazards model, and *P* < 0.05 considered statistically significant. All the data were represented as the mean ± SD of three replications.
